# Aggression and personal values in immigrant adolescents: A longitudinal examination of reciprocal associations

**DOI:** 10.1111/jora.70188

**Published:** 2026-04-23

**Authors:** Hanit Ohana, Seth J. Schwartz, Mark Van Ryzin, Adi Arden, Einat Elizarov, Maya Benish‐Weisman

**Affiliations:** ^1^ Paul Baerwald School of Social Work and Social Welfare The Hebrew University of Jerusalem Jerusalem Israel; ^2^ Department of Kinesiology and Health Education University of Texas at Austin Austin Texas USA; ^3^ College of Education, University of Oregon Eugene Oregon USA; ^4^ Department of Counseling and Human Development University of Haifa Haifa Israel

**Keywords:** adjustment, aggression, cultural context, immigrant youth, personal values

## Abstract

This longitudinal study investigates the bidirectional relationship between personal values and aggressive behavior among immigrant adolescents from the Former Soviet Union residing in Israel. Using a 4‐wave cross‐lagged latent difference score modeling approach over 2 years, we examined the reciprocal associations between personal values and aggressive behaviors over time. The sample included 180 adolescents (mean age = 14.36 years, *SD* = 1.35; 44.5% girls) and their primary caregivers, with youth reporting on personal values and both youth and parents reporting on adolescent aggression. Separate models were estimated for youth‐ and parent‐reported youth aggression. Our findings indicated that immigrant youth aggression predicted changes in personal values over time, but not vice versa. For youth‐reported models, adolescents' aggressive behavior predicted increases in self‐enhancement and openness to change values and decreases in self‐transcendence and conservation values. For parent‐reported models, youth aggressive behavior predicted increases in youth self‐enhancement values over time. These results highlight the asymmetry in the value‐behavior dynamic during adolescence, particularly as immigrant youth navigate and integrate into a new socio‐cultural environment. Our findings highlight the role of behavioral adaptation in shaping value systems during adolescence, offering insights into mechanisms underlying immigrant youth adjustment. Our findings emphasize the importance of targeting aggressive behaviors in interventions to foster adaptive values and enhance social integration.

## INTRODUCTION

Aggression involves behaviors aimed at intentionally causing harm to others (Fahy et al., 2016) and includes both physical (e.g., fighting, threatening) and social/relational (e.g., social exclusion, rumor‐spreading) forms, which frequently co‐occur in adolescence (see analytic review Card et al., 2008; Slawinski et al., 2019). During this period, aggression represents a prevalent concern, with short and long‐lasting effects on adolescents' mental and social well‐being (Arseneault, 2018; Nasaescu et al., 2020). The increasing prevalence of youth aggression, highlighted by UNICEF (2022), further emphasizes the urgent need to understand its antecedents and correlates, particularly among vulnerable groups such as immigrant adolescents facing sociocultural adjustment challenges (Vos et al., 2021). These youth face the dual challenge of adapting to a new culture while navigating the developmental complexities of adolescence (Ausubel, 2002; Forster et al., 2015; Smetana et al., 2006).

Research indicates that personal values play a key role in shaping behavior, serving as motivational principles that bridge internal drives with external actions (Schwartz, 1992, 2012). Personal values are defined as broad, trans‐situational goals that serve as guiding principles in people's lives, expressing what individuals regard as important and desirable (Schwartz, 2012). As guiding standards, they influence how adolescents behave and perceive the world (Feather, 1995), for example, by prioritizing concern for others (self‐transcendence) or emphasizing achievement and power (self‐enhancement), thereby impacting their interactions with the social environment. Extensive research supports the role of values in shaping social behaviors (for a review, see Roccas & Sagiv, 2010), particularly aggression during adolescence (e.g., Benish‐Weisman, 2015, 2019; Benish‐Weisman et al., 2017; Benish‐Weisman & McDonald, 2015).

Adolescence is also a period of developmental reorganization, during which both personal values and aggressive behavior may undergo meaningful change. While personal values tend to consolidate as part of identity development, they may shift in response to life transitions and developmental challenges (Bardi & Goodwin, 2011; Daniel et al., 2025; Daniel & Benish‐Weisman, 2019; Vecchione et al., 2020). Similarly, patterns of aggression may fluctuate or stabilize depending on adolescents' social environments and adaptation demand (Huesmann et al., 1984; Lansford, 2018). These processes highlight the importance of examining not only the mutual association between values and aggression, but also how each construct may change over time.

Although numerous studies have shown how changes in personal values predict future behavioral changes (Benish‐Weisman, 2015; Vecchione et al., 2016), emerging evidence suggests a reverse pathway: behavioral changes can also influence personal values, particularly during significant environmental transitions (Bardi & Goodwin, 2011). From a developmental and acculturative stress perspective (e.g., Jugert & Titzmann, 2020; Schwartz et al., 2010; Titzmann & Lee, 2018), adolescence is a critical period in which identity formation, autonomy, and peer belonging are especially salient, and these normative tasks become increasingly complex under conditions of migration‐related stress. For immigrant adolescents, who often experience both cultural dissonance and identity conflicts (Kanwal, 2022), aggressive behaviors may serve as a coping strategy—a way to assert autonomy, manage social exclusion, or resist constraints in a new cultural environment (Berry, 2006; Compas et al., 2001; Moffitt, 2017). Although not adaptive in the long term, such behaviors may arise in response to acculturative stress when more constructive strategies are unavailable. These patterns may, over time, contribute to shifts in adolescents' personal values. At the same time, shifts in their values may also influence their aggressive behaviors as they continue to adjust to their surroundings. However, the potentially bidirectional relationship between aggression and values over time, particularly among immigrant youth, remains poorly understood.

Unlike previous studies that primarily focused on concurrent associations between values and behavior (e.g., Benish‐Weisman & McDonald, 2015; Knafo et al., 2008) or explored reciprocal relationships among non‐immigrant adolescents using three waves, relying on adolescent self‐reports or peer nominations of aggression rather than incorporating parental perspectives (e.g., Benish‐Weisman, 2015; Vecchione et al., 2016), the present study bridges these gaps by examining the bidirectional relationship between self‐reported and parent‐reported aggression and values among immigrant adolescents using a four‐wave longitudinal design. It examines how aggressive behavior and values dynamically influence each other over time. Further, to detect meaningful developmental change while remaining sensitive to short‐term fluctuations, the study employed assessments spaced six months apart (Schwartz et al., 2016)—a design particularly well‐suited for capturing change during a period of heightened value and behavioral reorganization, especially in the context of adolescence (Vecchione et al., 2020) and recent immigration (Bardi & Goodwin, 2011; Schwartz et al., 2016; Tartakovsky & Schwartz, 2001). By capturing both directions of influence, the study offers a more nuanced understanding of the interplay between behavioral adaptation or maladaptation and value change amid the complex cultural transitions that characterize immigrant adolescence.

### Personal values and aggression

**FIGURE 1:**
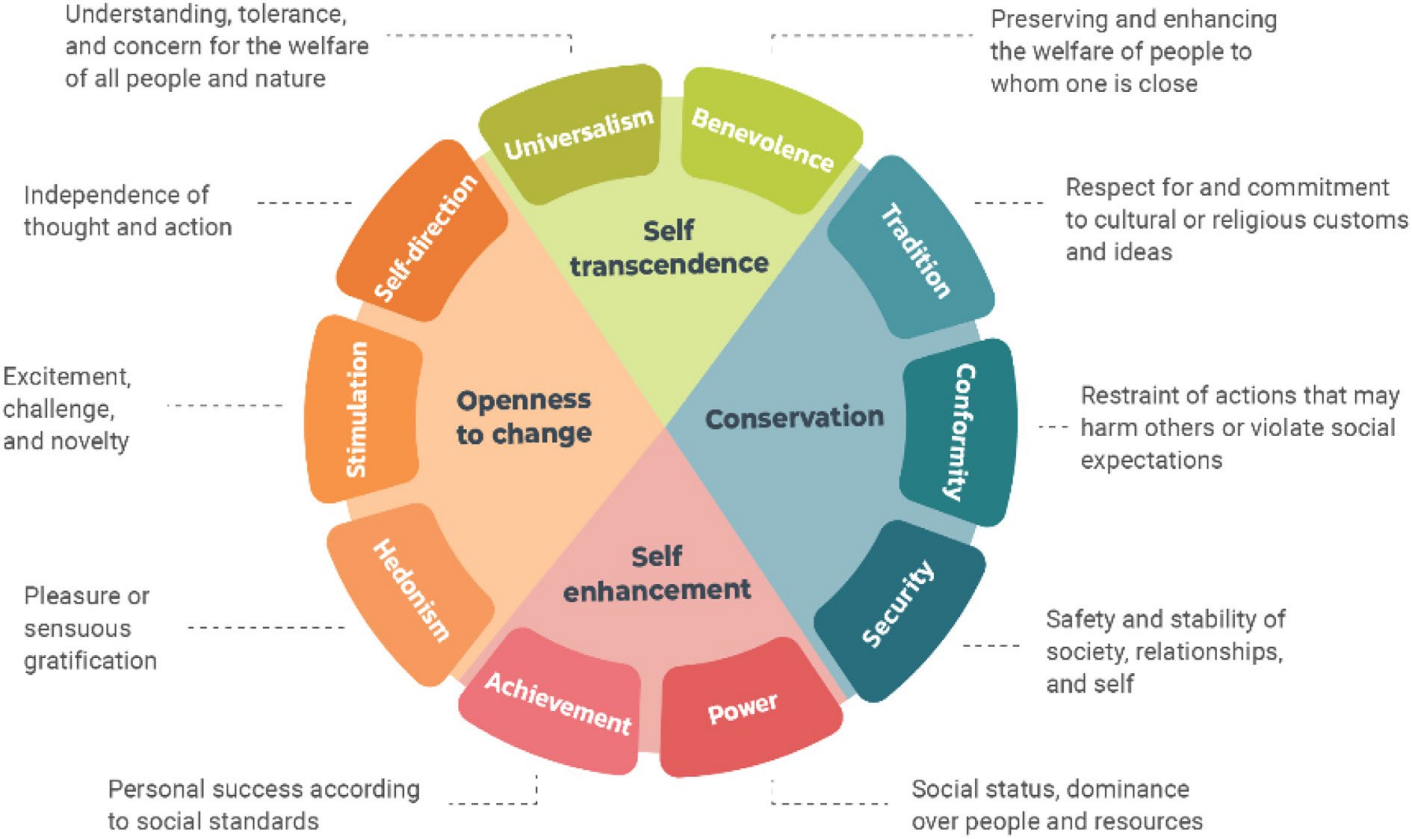
The Schwartz basic values model (adapted from Benish‐Weisman, 2024).

Personal values offer a framework for understanding why adolescents act the way they do, shaping their actions and choices by reflecting their priorities and motivations (Feather, 1995). Values guide decisions and behaviors, helping individuals achieve their goals, align with their sense of self, and maintain a sense of emotional well‐being (Sagiv & Roccas, 2021; Schwartz, 1992). By shaping what individuals consider important, values influence how they interpret and respond to social and cultural situations. Schwartz's (1992, 2012) theory of personal values provides a comprehensive model by organizing values along two bipolar dimensions: self‐enhancement versus self‐transcendence, and openness to change versus conservation (see Figure 1; Benish‐Weisman, 2024). This model provides insight into how different values influence individual behaviors and attitudes across various cultural and social contexts. According to Schwartz's theory, adolescents who prioritize power and personal success, such as self‐enhancement values, may encourage aggressive behaviors. In contrast, adolescents who attribute importance to care for others (self‐transcendence values) may be less likely to endorse or display aggression (Benish‐Weisman, 2019). The second value dimension, openness to change versus conservation, further shapes how individuals respond to their environments. Values that emphasize openness to change—such as stimulation and self‐direction—encourage flexibility, independence, and exploration. During adolescence, endorsing these values may heighten the likelihood of aggressive behaviors. A strong focus on novelty and autonomy often fuels sensation seeking and boundary‐testing, which are especially pronounced in peer groups where social hierarchies and norms are actively negotiated. Pursuit of new experiences in these contexts can involve risk‐taking, challenging authority, or experimenting with unconventional behaviors, all of which may escalate into aggressive interactions with peers (Benish‐Weisman, 2019; Benish‐Weisman & McDonald, 2015; Ungvary et al., 2018). Conversely, conservation values, which include tradition, conformity, and security, promote stability and adherence to social norms, often reducing the likelihood of aggressive responses as individuals are more focused on maintaining order and harmony (Schwartz, 1992, 2012). Research has supported the links between self‐enhancement and openness to change values with increased aggression (e.g., Benish‐Weisman, 2015; Benish‐Weisman et al., 2017; Danioni & Barni, 2019). Similarly, self‐transcendence and conservation values have been linked with reduced aggressive actions (Aquilar et al., 2018; Benish‐Weisman, 2015; Danioni & Barni, 2019). In the context of immigration, understanding how these values relate to behavioral adaptations is crucial yet remains underexplored, particularly given the unique social adjustment challenges faced by immigrant youth (Ohana et al., in press). Hence, the present study focuses on the dynamic relationship between values and aggressive behavior in immigrant youth, while also exploring the mutual influence between aggressive behavior and value change.

### How does behavior relate to future personal values among immigrant youth?

Although research and theories typically highlight how values guide behavior (e.g., Arieli et al., 2014; Benish‐Weisman, 2015; Maio et al., 2009), some evidence also points to instances where behavior itself initiates changes in values (e.g., Bardi & Goodwin, 2011; Benish‐Weisman, 2015; Vecchione et al., 2016). This reverse influence of behavior on values can be understood through the impact of environmental changes and the resulting need for individuals to align their actions with their beliefs. Environmental changes, especially during transitions to new settings, expose individuals to new norms and expectations that necessitate behavioral adaptations (Bardi & Goodwin, 2011). For immigrant youth, these transitions often coincide with adolescence, a time when peer influences are particularly strong (Jugert & Titzmann, 2020; Laursen & Veenstra, 2021). Pressures to adapt to new cultural contexts and satisfy developmental tasks such as identity formation, which involves establishing peer relationships and gaining social acceptance (Erikson, 1968; Smetana et al., 2006), may lead these adolescents to adopt behaviors, such as aggression, that contradict their pre‐existing values in order to fit in (Bardi et al., 2009; Rokeach, 1973).

Such behavioral changes can create a misalignment between existing values and new actions (Bardi & Goodwin, 2011; Rokeach, 1973). Driven by a need for self‐consistency, individuals may experience cognitive dissonance, prompting them to adjust their values to better align with their behavior (Festinger, 1962; Hogg & Vaughan, 2021). Self‐perception theory elaborates on this, suggesting that observing one's own behavior can lead adolescents to reassess their values, attributing these new behaviors to evolved personal standards (Bem, 1967; Rachlin, 2002). Immigrant adolescents are often found to experience more psychological, social, and educational challenges compared to their non‐immigrant counterparts, positioning them as a high‐risk group (Janssen et al., 2004; Mirsky, 2009; Oppedal & Røysamb, 2004; Slonim‐Nevo et al., 2006). These challenges may lead *some* immigrant youth to engage in aggressive behaviors as a coping strategy during adolescence (Moffitt, 2017). However, these responses are not uniform—while some adolescents may increase in aggression over time, others may remain stable, or show decreases. Over time, such behaviors could shape shifts in personal values, as adolescents adjust their value systems to align with their actions (Bem, 1967; Benish‐Weisman, 2019). The present study was designed to investigate whether and how these behavioral changes, particularly aggressive behaviors, influence changes in personal values among immigrant youth.

### The present study

This study extends the existing theoretical frameworks on the values‐behavior link proposed within Schwartz's (1992, 2012) basic values theory, and the behavior‐values link suggested by self‐perception theory (Bem, 1967) through exploring the bidirectional relationship between behavior and values within the context of social adjustment challenges during adolescence. Unlike previous studies that have primarily focused on concurrent or unidirectional influences of values on behavior, we suggest that reciprocal relations exist between values and behavior. To investigate these potential bidirectional associations, we examined the relationships between key values—including self‐enhancement, self‐transcendence, openness to change, and conservation—and aggressive behavior among immigrant adolescents.

We focused on immigrant adolescents from the Former Soviet Union (FSU) residing in Israel, a group facing distinct sociocultural and developmental challenges (Elizarov et al., 2023; Lee et al., 2022; Mirsky, 2012; Tamir & Ein‐Tal, 2025; Yakhnich et al., 2025) that may shape both their value development and behavioral responses within a broader integration context. Since the early 1990s, over one million immigrants from the FSU have arrived in Israel, forming one of the country's largest and most influential immigrant communities (Tartakovsky, 2025). Despite high levels of education and professional experience, many FSU immigrants experience downward social mobility, cultural dissonance, and limited integration into Israeli society (Remennick, 2019; Tamir & Ein‐Tal, 2025).

Research consistently shows that FSU immigrant youth are more likely to exhibit school maladjustment, including high rates of truancy, dropout, substance use, and aggressive behavior (Mirsky, 2012; Tamir & Ein‐Tal, 2025; Yakhnich et al., 2025). Social adjustment is often strained by peer rejection and ethnic segregation, with FSU youth reporting exclusion from Israeli‐born peer groups and frequent intergroup conflict, often escalating to aggressive behaviors (e.g., Tamir & Ein‐Tal, 2025; Yakhnich et al., 2025). In such contexts, aggressive behavir may serve as a coping strategy, taking both physical forms (e.g., verbal insults, threats, or confrontations) and social forms (e.g., social exclusion or spreading rumors). These behavioral patterns may reflect or coincide with shifts in personal values. Among FSU youth, tensions may arise as their personal values evolve in response to shifting social environments as they navigate peer dynamics and sociocultural adaptation. Given that FSU immigrant youth comprise approximately 12.1% of Israel's population (Central Bureau of Statistics, 2021), understanding their psychosocial development is vital for designing culturally responsive school‐based interventions, improving access to mental health services, and guiding policies that support immigrant youth integration.

Values predicting changes in aggressive behavior.

Guided by Schwartz's theory of basic values (1992, 2012) and prior research on aggression and adolescent value orientations (e.g., Benish‐Weisman, 2015, 2019; Danioni & Barni, 2019), we hypothesized that personal values among immigrant adolescents would be associated with changes in aggressive behavior. Specifically, *self‐enhancement* values, which emphasize personal success, dominance, and power, are expected to be associated with increases in aggression, as adolescents may assert control or gain status through confrontational behavior. *Openness to change* values, such as stimulation and self‐direction, reflects a desire for novelty and autonomy, and may also be associated with increased aggression through sensation‐seeking and norm‐challenging behaviors. In contrast, *self‐transcendence* values, which include benevolence and universalism, promote concern for others and are expected to be associated with decreases in aggression. Similarly, *conservation* values, including tradition, conformity, and security, encourage self‐restraint and adherence to social norms and are thus expected to be associated with decreases in aggression.

Aggressive behavior predicting changes in personal values.

For the reverse direction, we drew on self‐perception theory (Bem, 1967) and research on behavior‐driven value change (e.g., Bardi & Goodwin, 2011; Benish‐Weisman, 2015; Vecchione et al., 2016), and hypothesized that aggressive behavior would be associated with changes in personal values. In addition, building on developmental and acculturative stress frameworks (e.g., Jugert & Titzmann, 2020; Schwartz et al., 2010; Titzmann & Lee, 2018), we propose that immigrant adolescents who engage in aggressive behavior may subsequently adjust their personal values to reduce internal dissonance and maintain a coherent self‐concept. Specifically, aggressive behavior is expected to be associated with increased endorsement of self‐enhancement and openness to change values, and decreased endorsement of self‐transcendence and conservation values.

To examine these hypotheses, we employed cross‐lagged latent difference score modeling (CLDSM; McArdle, 2009) to track reciprocal changes across four waves, examining relationships of the four value dimensions with aggression. Unlike alternative approaches such as growth curve models (Curran et al., 2010) or RI‐CLPM (Hamaker et al., 2015), which do not explicitly estimate latent change, this approach allows us to capture both the direction and magnitude of these changes while also accounting for baseline levels of values and aggressive behavior.

Evidence of aggression in prior studies of FSU immigrant youth has relied primarily on adolescents' self‐reports (Tamir & Ein‐Tal, 2025; Yakhnich et al., 2025) and, in some cases, teacher and school records (Mirsky, 2012). Both approaches have limitations: youth may minimize socially undesirable behaviors, whereas institutional reports may be influenced by stereotypes portraying FSU youth as disruptive (Mirsky, 2012). By incorporating parents' perspectives alongside youth self‐reports, our study extends this literature in important ways. Parent reports are particularly valuable in immigrant families, where peer nominations are less feasible due to language barriers, social segregation, and high mobility in school contexts (e.g., Tamir & Ein‐Tal, 2025; Yakhnich et al., 2025), and where both self‐ and teacher‐reports may be subject to bias (Mirsky, 2012). To address these limitations, we include both parent and youth reports of aggression to capture distinct perspectives on adolescents' behavior. Prior research has consistently documented discrepancies between these reports, reflecting differences in behavioral context and interpretation rather than measurement error (Lansford, 2018; Yang et al., 2021). We therefore estimated separate models for each informant, allowing us to examine aggression–value associations across perspectives.

## METHODS

### Participants

This longitudinal study used data collected at four waves over a span of 2 years. The sample comprised 180 adolescent immigrants from the FSU and their primary parent figures, who had moved to Israel within the 5 years preceding the initial assessment (T1). Adolescents' (55.5% boys) mean age at T1 was 13.70 (*SD* = 1.35), ranging from 11 to 17. The majority (72.1%) were between 13 and 15 years old, reflecting a sample concentrated in early to mid‐adolescence. Most participating parents (87.6%) were mothers, with an average age of 41.56 years (*SD* = 5.30 years). The majority of parents (81.7%) held at least a bachelor's degree. Marital status among the parents varied, with 77.5% married, 16.8% divorced, and the remainder separated or never married. The participant families primarily originated from Russia (61.5%), Ukraine (33.2%), and Belarus (5.3%), and had lived in Israel for an average of 2.71 years (*SD* = 1.61 years) by T1. Nearly half of the families (48.5%) reported monthly incomes up to 10,000 New Shekels (approximately US$1500 to US$3000), which is considerably below the 2021 national average income of 19,916 New Shekels (Israel Central Bureau of Statistics, 2021).

### Procedure

Data collection for Time 1 took place from June to December 2020, with three subsequent assessments occurring every 6 months until June 2022. Participants were recruited through social media, word‐of‐mouth, and referrals. Researchers contacted interested families to explain the study's goals and procedures. After obtaining informed consent, parents and adolescents were provided with a link to complete the online assessment battery. This battery was completed at each of the four timepoints. Upon completion of each assessment, parents and adolescents received gift cards valued at $23 and $10, respectively. The study protocol was approved by the ethics committee at the Hebrew University of Jerusalem (approval number: 133/20).

### Measures

All measures were back‐translated into Russian or Hebrew and administered in participants' preferred language.

#### Personal values

Youth values were assessed using the *Portrait Values Questionnaire* (PVQ; Schwartz et al., 2001). The PVQ has been extensively validated and widely applied in adolescent samples across both cross‐sectional and longitudinal studies (Benish‐Weisman, 2015; Bubeck & Bilsky, 2004; Knafo et al., 2008; Schwartz et al., 2001; Vecchione et al., 2020). This tool includes short verbal portraits of 40 people, gender‐matched with the respondent. Each verbal portrait describes one's goals, aspirations, or wishes and implicitly indicates a specific core value. Participants are asked to answer how similar they think they are to each portrait provided, ranging from not like me at all (1) to very much like me (6). Following Schwartz (1992), a standard procedure was used to control for response tendency. To control for respondents' scale use, individuals' responses were centered on their average rating across all 40 items (Schwartz, 1992). We then computed average scores for each of the four higher‐order value groups by averaging the centered items associated with each value domain. The first value group, self‐enhancement values (power and achievement), focuses on promoting the self and controlling others. For example, “It is important to her to be in charge and tell others what to do. She wants people to do what she says” ($\alpha_{T1}$ = .83, $\alpha_{T2}$ = .85, $\alpha_{T3}$ = .83, $\alpha_{T4}$ = .82). The second value group, self‐transcendence values (benevolence and universalism), emphasizes care for others' rights and welfare. A sample item is “It is important to her to respond to the needs of others. She tries to support those she knows” ($\alpha_{T1}$ =.78, $\alpha_{T2}$ = .78, $\alpha_{T3}$ = .79, $\alpha_{T4}$ = .83). The third value group, openness to change values, encompasses three key attributes: self‐direction, stimulation, and hedonism. This construct refers to an individual's positive orientation toward change through accepting and adapting new ideas, experiences, and actions. For example, “Thinking up new ideas and being creative is important to her. She likes to do things in her own original way” ($\alpha_{T1}$ = .82, $\alpha_{T2}$ = .73, $\alpha_{T3}$ = .79, $\alpha_{T4}$ = .82). The fourth value group, conservation values (tradition, conformity, and security) signify a commitment to preserving social order and upholding the existing status quo. A sample item for these values is “It is important to her always to behave properly. She wants to avoid doing anything people would say is wrong” ($\alpha_{T1}$= .76, $\alpha_{T2}$ = .77, $\alpha_{T3}$ = .78, $\alpha_{T4}$ = .83).

#### Aggression

Youth's aggressive behavior was assessed using both adolescent self‐reports and parent reports, capturing a broad range of *physical* (e.g., fighting, threatening) and *social/relational* (e.g., excluding peers, spreading rumors) behaviors. Although these subtypes differ in expression, they are highly correlated and frequently co‐occur during adolescence (Card et al., 2008, meta‐analytic *r* = .76; see also Slawinski et al., 2019). Accordingly, we used a composite score to capture overall aggressive behavior rather than analyzing subtypes separately.

Youth‐reported aggression was measured using 11 items from the Social/Relational Aggression subscale of the Subtypes of Antisocial Behavior Questionnaire (STAB; Burt & Donnellan, 2009; see Table S2), assessing aggressive behaviors such as “Excluded someone from group activities when angry with them; was rude towards others; tried to hurt someone's feelings”, referring to the 6 months prior to assessment. Responses were rated on a 3‐point scale: “not true” (0), “sometimes true” (1), or “often/very true” (2) ($\alpha_{T1}$ = .75, $\alpha_{T2}$ = .84, $\alpha_{T3}$ = .83, $\alpha_{T4}$ = .84). At each wave, a composite aggression score was computed as the mean of the 11 items (minimum 8 items required), with higher scores indicating greater youth‐reported social/relational aggression. The STAB has been used with adolescent populations (Nnadozie et al., 2022), supporting its relevance for use in our sample.

*Parent‐reported* youth aggression was assessed using six items from the Revised Behavior Problem Checklist (RBPC; Quay & Peterson, 1987) specifically designed to measure aggressive behaviors aimed at harming others. Items were initially selected based on their theoretical relevance and content validity, which was established through their alignment with constructs assessed in youth self‐reports of aggressive behaviors (Burt & Donnellan, 2009). Content validity ensured that the selected items captured key aspects of aggressive behaviors, such as “Fights; bullies; blames others; denies own mistakes.” Construct validity was subsequently evaluated through confirmatory factor analysis (CFA) to determine whether the items consistently loaded onto the aggression factor and accurately measured the construct. The results demonstrated that at T1, sex items loaded strongly onto the aggression factor, with standardized factor loadings ranging from .53 to .67 (see Table S1). Responses were rated on a 3‐point scale: no problem/don't know (0), mild problem (1), and severe problem (2) ($\alpha_{T1}$ = .80, $\alpha_{T2}$ = .81, $\alpha_{T3}$ = .78, $\alpha_{T4}$ = .74).

### Treatment of missing data and plan for analysis

Of the 180 participating families at the first wave, 139 families (78%) participated in the second wave, 128 families (71%) participated in the third wave, and 125 families (69%) participated in the fourth wave. The percentage of missing data ranged between 12.9% and 20.0% at T1, between 31.6% and 36.9% at T2, between 40.4% and 46.2% at T3, and between 37.3% and 46.2% at T4. Little's missing completely at random (MCAR) test was significant, χ^2^ (716) = 785, *p* = .04, indicating that the variables were not MCAR. However, the normed chi‐square (χ^2^/df = 1.10) was well below the recommended threshold of 2.0, suggesting that the departure from MCAR was minor. An attrition analysis comparing participants who remained in the study to those who dropped out after T1 revealed no significant differences across key study variables (see Table S3). Further analyses indicated no significant correlations between missingness and key demographic variables, including youth gender (*r* = .12, *p* = .10), age (*r* = −.02, *p* = .84), grade (*r* = −.08, *p* = .25), or time since immigration (*r* = .04, *p* = .75). In addition, correlations between missingness and study variables ranged from −.09 to .14, none of which were statistically significant. These results provide evidence against systematic bias in the missing data.

**FIGURE 2:**
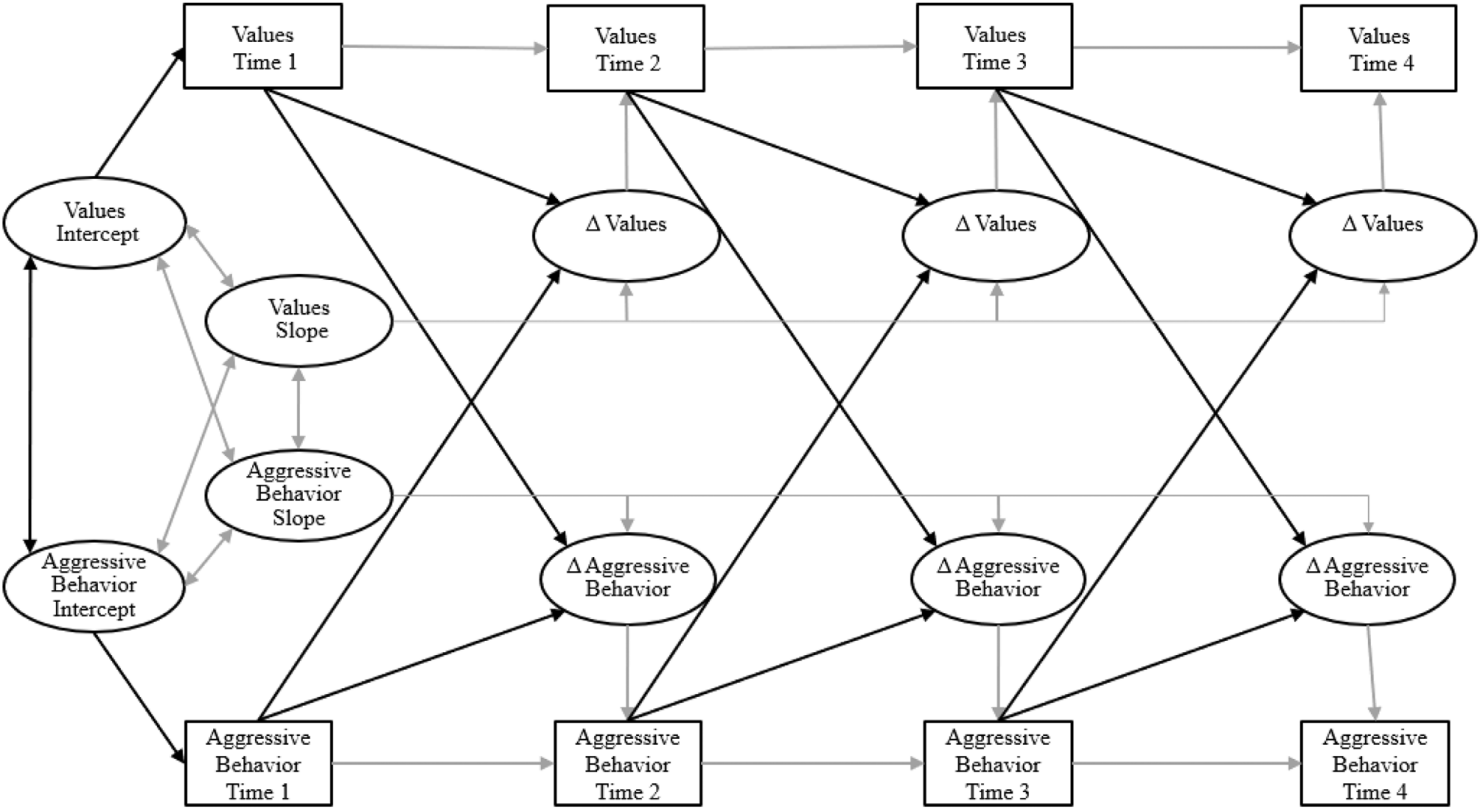
Theoretical model: cross‐lagged difference score model linking immigrant youth values and aggression at time 1 to time 4.

All models were therefore estimated using Maximum Likelihood (ML) estimation with robust standard errors, which can provide unbiased estimates in the presence of missing data and/or non‐normal distributions (Enders & Bandalos, 2001). We first evaluated the means and variances for intercept and slope terms to identify significant initial levels and changes over time in values and aggressive behavior (see Tables S4 and S5). Then, to evaluate our hypotheses, we employed CLDSM, which decomposes change over time into a series of segments representing the amount of change from one measurement wave to the next. Difference scores are then used in an autoregressive cross‐lagged framework to assess the degree to which each variable influences change in the other over time (McArdle, 2009). These difference scores are latent constructs that represent the amount of change between adjacent waves, which enables us to obtain accurate assessments of the influence of one variable (e.g., aggressive behavior) on the net change in another variable (e.g., self‐enhancement) while controlling for the influence of baseline levels of both variables (McArdle, 2009). Cross‐lagged difference score models also enable us to evaluate the amount of change over time in each construct, providing important context. In this way, these models combine the functionality of cross‐lagged models and growth curve models into a single cohesive framework. We provide an example of the theoretical model in Figure 2, where we estimate the relationship between aggressive behavior and values and evaluate how each variable predicts subsequent change in the other.

**FIGURE 3:**
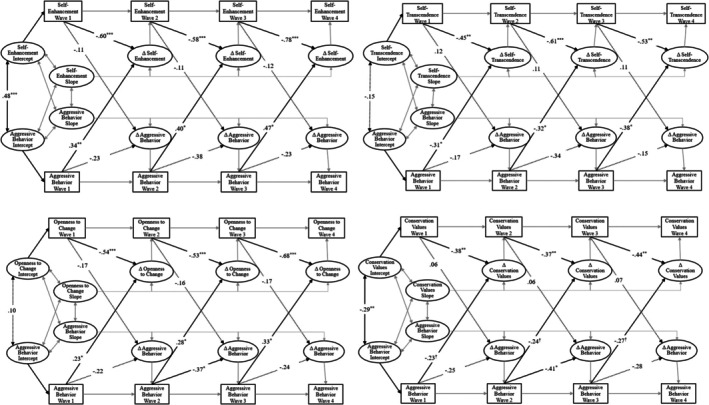
Cross‐lagged difference score models with youth‐reported aggressive behavior. *p < .05. **p < .01. ***p < .001.

**FIGURE 4:**
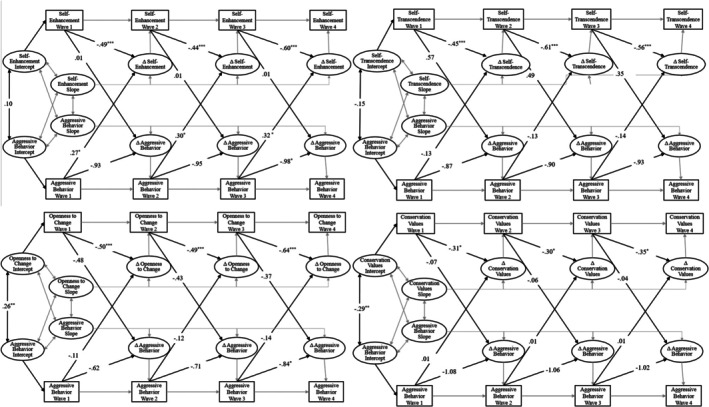
Cross‐lagged difference score models with parent‐report of youth aggressive behavior. *p < .05. **p < .01. ***p < .001.

We fit our cross‐lagged difference score model using Mplus 7.4 (Muthén & Muthén, 1998–2015) and ML estimation with robust standard errors. For each model (see Table 2), we provide standard measures of fit, including the chi‐square (χ^2^), comparative fit index (CFI), non‐normed or Tucker‐Lewis index (TLI), and root mean square error of approximation (RMSEA). CFI and TLI values greater than .90, and RMSEA values below .05, indicate good fit (Schwartz et al., 2010). We used all available data in each analysis as per standard practice with ML (Schafer & Graham, 2002). We fit separate models for both youth‐reported and parent‐reported youth aggressive behavior with youth self‐enhancement, self‐transcendence, openness to change, and conservation values, evaluating how each construct influences subsequent changes in the others over time. Models and results are presented in Figures 3 and 4. Given the broad age range of the sample (11–17 years), during which both adolescent values and aggression are likely to vary, and established gender differences in aggression, supplementary analyses controlled for age and gender; results remained consistent in direction and significance with the primary models.

## RESULTS

Descriptive statistics and correlations are presented in Table 1. Correlations between youth‐ and parent‐reported aggression were generally modest and time‐specific, with same‐wave associations reaching significance at Wave 3 only and additional cross‐wave associations observed (Table 1, see also Table S6). Gender‐specific descriptive statistics and correlations are provided in the (Tables S7a,b). Descriptive data for the means and variances of intercepts and slopes for each model component are presented in Supporting Information (Table S4 for youth‐report and Table S5 for parent‐report). Self‐enhancement, self‐transcendence, openness to change, and conversation values had significant intercepts and slopes, indicating a degree of change over time. Youth‐reported aggressive behavior also had a significant intercept but nonsignificant change over time (although there was significant variance in change over time, indicating that youth in the sample followed a variety of different trajectories). In contrast, parent reports of youth aggressive behavior had nonsignificant change over time and nonsignificant variance in change over time.

**TABLE 1:** Correlations, means, and standard deviations.

	Self‐E				Self‐T				Open				Con				Stab				Rbpc			
	1	2	3	4	5	6	7	8	9	10	11	12	13	14	15	16	17	18	19	20	21	22	23	24
1. Self‐E (W1)	—																							
2. Self‐E (W2)	.68***	—																						
3. Self‐E (W3)	.68***	.76***	—																					
4. Self‐E (W4)	.39***	.64***	.71***	—																				
5. Self‐T (W1)	−.55***	−.38***	−.38***	−.14	—																			
6. Self‐T (W2)	−.45***	−.57***	−.50***	−.37***	.61***	—																		
7. Self‐T (W3)	−.29**	−.33***	−.55***	−.39***	.60***	.64***	—																	
8. Self‐T (W4)	−.15	−.19	−.34***	−.42***	.46***	.52***	.67***	—																
9. Open (W1)	.23**	.28***	.28***	.12	−.32***	−.21**	−.21**	−.07	—															
10. Open (W2)	.19*	.21**	.17	.10	−.19*	−.28***	−.11	.02	.64***	—														
11. Open (W3)	.14	.19*	.19*	.05	−.12	−.13	−.24**	−.04	.56***	.63***	—													
12. Open (W4)	.11	.16	.18	.23**	−.03	−.13	−.09	−.17	.43***	.50***	.71***	—												
13. Con (W1)	−.53***	−.47***	−.47***	−.31***	−.04	.07	−.06	−.15	−.72***	−.50***	−.44***	−.39***	—											
14. Con (W2)	−.41***	−.57***	−.42***	−.32***	.03	−.04	−.13	−.26**	−.53***	−.67***	−.49***	−.36***	.72***	—										
15. Con (W3)	−.41***	−.45***	−.50***	−.30***	−.06	.04	−.12	−.22*	−.49***	−.52***	−.70***	−.59***	.75***	.75***	—									
16. Con (W4)	−.27**	−.41***	−.39***	−.56***	−.18	.02	−.11	−.24**	−.30***	−.39***	−.45***	−.70***	.57***	.59***	.74***	—								
17. Stab (W1)	.37***	.36***	.38***	.17	−.20**	−.17*	−.16	−.01	.10	.12	.17	.08	−.20*	−.24*	−.27**	−.16	—							
18. Stab (W2)	.26**	.38***	.43***	.31**	−.01	−.18*	−.17	−.05	.08	.17*	.13	.10	−.22**	−.30***	−.28**	−.25*	.57***	—						
19. Stab (W3)	.19*	.24**	.36***	.31**	.10	−.02	−.08	−.14	−.04	.00	.10	.19	−.17	−.18	−.25**	−.23*	.51***	.61***	—					
20. Stab (W4)	.21*	.27**	.34***	.39***	.05	−.19	−.24*	−.28**	−.09	.03	.05	.18	−.11	−.08	−.12	−.21*	.39***	.53***	.61***	—				
21. Rbpc (W1)	.03	.13	.15	.18	−.09	−.19*	−.09	−.15	.19*	.17*	.05	−.01	−.08	−.07	−.08	.00	.02	.02	.07	.10	—			
22. Rbpc (W2)	.09	.19*	.21*	.17	−.07	−.16	−.09	−.12	.23**	.11	.03	−.08	−.21*	−.14	−.11	.01	.07	.09	.11	.02	.67***	—		
23. Rbpc (W3)	.15	.17	.25**	.18	−.01	−.28**	−.16	−.13	−.02	.01	−.03	.06	−.09	.01	.07	−.07	−.06	.11	.27**	.19	.58***	.71***	—	
24. Rbpc (W4)	.17	.17	.28**	.20*	−.06	−.15	−.18	−.02	.04	.01	−.05	−.03	−.10	−.06	−.06	−.11	.18	.35***	.24*	.13	.40***	.56***	.41**	—
*N*	196	153	128	125	196	153	128	125	196	153	128	125	196	153	128	125	180	142	121	121	180	151	130	112
*M*	3.81	3.81	3.99	4.03	4.30	4.35	4.20	4.20	4.47	4.42	4.43	4.37	3.45	3.44	3.44	3.45	1.29	1.29	1.24	1.26	8.21	8.29	8.08	7.86
*SD*	.76	.82	.74	.73	.50	.50	.50	.45	.56	.49	.54	.50	.56	.52	.55	.58	.26	.31	.28	.28	1.94	1.98	1.82	1.54

Abbreviations: Rbpc, Parent‐reported youth aggression; Stab, Youth‐reported aggression.

* *p* < .05.

** *p* < .01.

*** *p* < .001.

### Youth‐reported aggressive behavior and values

Table 2 presents the model fit indices for youth‐reported and parent‐reported aggression and values over time, demonstrating that all models yielded good fit. The cross‐lagged difference score models with youth‐reported aggressive behavior (presented in Figure 3) indicated that self‐enhancement, self‐transcendence, openness to change, and conversation values all exhibited significant and negative autoregression coefficients, implying that high levels at one time point (e.g., at T1) predicted lower levels of subsequent change (e.g., from T1 to T2). In contrast, aggressive behavior exhibited no significant autoregression coefficients, indicating that behavior at one time point did not predict subsequent change (e.g., aggressive behavior at T1 did not predict change in aggression from T1 to T2). Controlling for autoregression, model estimates indicated significant associations between immigrant youth aggression and changes in personal values over time. Specifically, greater levels of youth‐reported aggressive behavior predicted subsequent increases in self‐enhancement values (β = .34 to .47, all *p* < .05) and openness to change values (β = .23 to .33, all *p* < .05). Conversely, aggressive behavior was predicted decreases in self‐transcendence values (β = −.31 to −.38, all *p* < .05) and conservation values (β = −.23 to −.27, all *p* < .10). In contrast, none of the value dimensions predicted change in youth‐report aggressive behavior across the four models.

**TABLE 2:** Model fit indices for youth‐reported and parent‐reported aggression and values over time.

Youth‐reported aggression models						Parent‐reported aggression models				
	χ^2^	*Df*	CFI	TLI	RMSEA (90% CI)	χ^2^	*Df*	CFI	TLI	RMSEA (90% CI)
Model 1 Aggression and self‐enhancement values	43.08	24	.96	.96	0.062 (0.030–0.091)	51.85	24	0.95	0.94	0.074 (0.046–0.102)
Model 2 Aggression and self‐transcendence values	45.11	24	0.95	0.94	0.065 (0.035–0.094)	45.16	24	0.95	0.95	0.065 (0.034–0.093)
Model 3 Aggression and openness to change values	29.96	24	0.99	0.98	0.035 (0.000–0.070)	58.73	24	0.93	0.92	0.083 (0.056–0.110)
Model 4 Aggression and conservation values	30.71	24	0.99	0.98	0.037 (0.000–0.071)	45.54	24	0.96	0.96	0.065 (0.035–0.094)

*Note*: CFI and TLI > 0.95 indicate excellent fit, while 0.90–0.95 suggest good fit. RMSEA <0.06 indicates excellent fit, and <0.08 suggests good fit. The cross‐lag difference score models examined longitudinal links between aggression and self‐enhancement, self‐transcendence, openness to change, and conservation values, as reported by youth and parents.

Abbreviations: χ^2^, Chi‐square goodness‐of‐fit statistic; CFI, comparative fit index; RMSEA, root mean square error of approximation (with 90% confidence intervals); TLI, Tucker–Lewis index.

### Parent‐reported aggressive behavior and values

As shown in Table 2, the cross‐lagged difference score models with parent‐reported youth aggressive behavior yielded adequate model fit. These models (presented in Figure 4), like youth‐reported models, indicated that self‐enhancement, self‐transcendence, openness to change, and conversation values all exhibited significant and negative autoregression coefficients, implying that high levels at one time point (e.g., at T1) predicted lower levels of subsequent change (e.g., from T1 to T2). In addition, similar to youth‐reported aggressive behavior, parent reports of youth aggressive behavior exhibited no significant autoregression coefficients, indicating that behavior at one time point did not predict subsequent change (e.g., aggressive behavior at T1 did not predict change in aggression from T1 to T2). Controlling for autoregression, model estimates indicated only one significant association between parent‐reported youth aggressive behavior and changes in personal values over time. Specifically, greater parent‐reported youth aggressive behavior predicted increases in self‐enhancement values (β = .25 to .30, all *p* < .05). No other cross‐lagged paths were significant.

Notably, although age and gender were not included as covariates in the main models, preliminary analyses indicated significant associations between age and youth‐reported aggression (*r* = .15, *p* = .039) and gender (1 = male; 2 = female) and parent‐reported aggression (*r* = .26, *p* < .001). These findings indicate that older adolescents reported higher levels of aggression and that girls were perceived by parents as displaying more aggressive behavior than boys in our sample. This pattern is broadly consistent with prior research on developmental and gender differences in aggression (Card et al., 2008; Slawinski et al., 2019), although these studies distinguish between aggression subtypes, whereas the present study relies on a composite measure of aggression. SES and time since immigration were unrelated to values or aggression and were therefore excluded. To assess robustness, we conducted supplementary analyses controlling for age in the youth‐reported models and gender in the parent‐reported models. Supplementary analyses controlling for age in the youth‐reported models and gender in the parent‐reported models yielded consistent results in direction and significance.

## DISCUSSION

This study was designed to investigate the reciprocal relationships between youth aggressive behavior and personal values over time, using both youth‐ and parent‐reported measures of aggression. We focused on immigrant adolescents navigating the socio‐cultural challenges of migration during adolescence.

Our findings indicated significant changes in values (self‐enhancement, self‐transcendence, openness to change, and conservation) across four waves, with higher initial levels (e.g., at T1) predicting smaller subsequent changes (e.g., from T1 to T2). The results are consistent with research suggesting that as values consolidate, they integrate into adolescents' broader identity systems, making them less likely to change (Cieciuch et al., 2016; Daniel et al., 2025; Daniel & Benish‐Weisman, 2019). Our findings indicate that migration during adolescence is associated with a more dynamic reorganization of values, driven by the dual demands of cultural adaptation (i.e., values change in response to life‐changing events; Bardi & Goodwin, 2011) and identity formation (Meeus, 2011). This process may enable immigrant youth to balance external acculturative pressures with internal needs for coherence, resulting in a more adaptive and resilient value system.

Aggression, based on both youth and parent reports, remained stable across four waves, with no significant mean change and no evidence of behavior at one time point predicting subsequent changes. This stability aligns with prior research on the consistency of aggression during adolescence (Huesmann et al., 1984; Lansford, 2018; Petersen et al., 2015) and may reflect its role as a coping mechanism for migration‐related stressors, such as cultural dissonance (Kanwal, 2022) or peer challenges (Strohmeier & Spiel, 2012). For instance, recent studies among immigrant youth from the FSU in Israel suggest that aggression may serve as a reactive strategy to defend one's social identity, assert autonomy, or cope with perceived marginalization in unfamiliar peer environments (Tamir & Ein‐Tal, 2025; Yakhnich et al., 2025).

### Reciprocal links between aggression and values over time in immigrant youth

Our findings supported the hypothesis that aggressive behavior predicts shifts in personal values over time (H2), but not the reverse pathway (H1). Specifically, higher levels of youth‐reported aggressive behavior were associated with increases in self‐enhancement and openness to change values, and with decreases in self‐transcendence and conservation values. However, when youth aggression was reported by parents, greater levels of aggression only predicted increases in self‐enhancement values. For both reporters, no evidence emerged to suggest that values influenced subsequent changes in aggressive behavior, suggesting that this association is largely unidirectional during youth's transition to a new country.

This asymmetry aligns with emerging evidence suggesting that behaviors, particularly in the context of migration, can serve as catalysts for changes in values (e.g., Bardi & Goodwin, 2011). Our results differ from those of Benish‐Weisman (2015), who examined Israeli adolescents during the transition from middle to high school and, by estimating directional paths between constructs, found that self‐enhancement values predicted higher subsequent aggression, whereas aggression evidenced reciprocal associations with self‐transcendence values across time. By contrast, in our study of immigrant adolescents, where changes were modeled directly, aggression predicted shifts across all four value domains, whereas values did not predict later aggression. Theoretically, this divergence suggests that in normative school transitions, values may exert a guiding influence on behavior. However, in the presence of migration‐related stressors, aggression may instead function as a coping strategy that reorganizes value priorities in response to the challenges of adaptation. In the present study with recent immigrant youth, adolescents who acted aggressively may have attributed these behaviors to values such as self‐enhancement and openness to change, aligning their value systems with their actions to reduce cognitive dissonance (Bardi & Goodwin, 2011; Bem, 1967; Festinger, 1962). For immigrant youth, who often encounter cultural dissonance, peer rejection, and the pressures of navigating social acceptance in a new environment, such aggressive behaviors may simultaneously function as adaptive responses to their unique challenges (Jugert & Titzmann, 2020; Laursen & Veenstra, 2021). Aggression in this context may serve as a strategic, goal‐directed behavior aimed at asserting dominance, gaining peer recognition, or securing social status—particularly in competitive or exclusionary environments. These behaviors may align with self‐enhancement values that emphasize personal success and power. Similarly, aggression may facilitate openness to change by challenging traditional norms and promoting adaptability. Migration exposes immigrant youth to new cultural expectations that require flexibility and independence. Aggression may reflect a willingness to defy conformity, embrace novel experiences, and seize social opportunities, tendencies often associated with sensation seeking, aligning with values of exploration, autonomy, and adaptability. At the same time, the immediate demands of survival and social integration may lead one to deprioritize values such as self‐transcendence, which emphasize empathy and social harmony, and conservation, which focuses on stability and tradition. As stated in the introductory section, aggressive behavior often exerts a negative impact on adolescents and their social environments. However, we suggest that, in a new and uncertain environment, aggression may reflect a pragmatic response to external pressures, reinforcing values that facilitate immediate adaptation at the expense of those promoting long‐term social harmony.

The developmental context of adolescence further amplifies the effects of aggressive behavior on value shifts in the context of migration. Adolescence is a period marked by identity formation and the establishment of peer relationships (Erikson, 1968; Smetana et al., 2006), characterized by biological, social, and psychological changes that shape behavior and values. Building on developmental and acculturative stress frameworks (e.g., Jugert & Titzmann, 2020; Schwartz et al., 2010; Titzmann & Lee, 2018), these developmental changes may become particularly complex for immigrant adolescents, as they intersect with the demands of acculturation and intensify challenges related to cultural adaptation. Migration during this stage compounds these pressures, and aggressive behaviors may function as a developmental strategy to assert independence and solidify a social identity within their new cultural context. These behaviors align with self‐enhancement and openness to change values by enabling adolescents to explore their autonomy, challenge traditional norms, and navigate new social dynamics. Conversely, developmental pressures during adolescence may deprioritize self‐transcendence and conservation values, as the focus shifts to immediate developmental tasks such as gaining social acceptance and achieving a sense of agency in the face of environmental upheaval.

Importantly, the lack of evidence supporting the reverse pathway—where values would predict changes in aggressive behavior—highlights the primacy of behavior in driving value change among immigrant youth. Although values are traditionally seen as guiding principles that influence behavior (Schwartz, 1992, 2012), our findings suggest that, under conditions of environmental and developmental flux, such as migration during adolescence, behaviors may play a more immediate role in adapting to external pressures. In a new environment, adolescents might not be able to act according to their values, as they may be subject to external forces such as social pressure. Behaviors tend to shift more rapidly in response to environmental demands, whereas values are more stable and adapt gradually over time (Benish‐Weisman, 2019; Lee et al., 2020). This asymmetry may reflect the urgent need for behavioral adaptations in high‐stress contexts, where aggressive actions serve as immediate strategies to navigate social and cultural challenges. In turn, these behaviors might shape value systems through mechanisms such as self‐perception (Bardi et al., 2009; Bem, 1967) and cognitive dissonance (Bardi & Goodwin, 2011; Festinger, 1962), prompting adolescents to align their values with their actions. Our findings underscore the unique vulnerability of immigrant youth, for whom behavioral adaptations may reflect efforts to reconcile internal and external demands, emphasizing the need for interventions targeting aggressive behaviors to foster long‐term value systems that prioritize social harmony and positive adjustment.

The finding that youth‐ and parent‐reported models differed also warrants consideration. Youth‐reported aggression predicted changes across all four value domains, whereas parent‐reported aggression was associated only with self‐enhancement. This divergence likely reflects the distinct perspectives each informant provides: adolescents' self‐reports capture internal motivations that are more directly tied to values (Schwartz, 1992), whereas parents' reports emphasize observable dominance‐related behaviors, which map more narrowly onto self‐enhancement (Lansford, 2018; Yang et al., 2021).

### Strengths, limitations, and future directions

Our study has several notable strengths. First, it advances the understanding of the reciprocal relationship between values and aggression among immigrant adolescents by employing a robust longitudinal design with four waves of data collection. Second, the use of CLDSM (McArdle, 2009) provides a powerful framework for examining the reciprocal relationships between values and aggressive behavior over time. Traditional models, such as the random intercept cross‐lagged panel model (RI‐CLPM; Hamaker et al., 2015), are increasingly used to isolate within‐person associations by removing between‐person variance. However, they do not model *change scores* directly. In contrast, CLDSM explicitly estimates latent change between time points, allowing us to examine how one construct predicts change in another over time. Because our primary interest was in understanding whether aggression predicts shifts in personal values (and vice versa), CLDSM offered the most appropriate framework. Furthermore, by modeling changes across four time points while controlling for baseline levels, this approach disentangled the dynamic interplay between values and aggression, illuminating how one domain influences the other, particularly during the critical period of adolescence within the context of migration.

Third, self‐reports of aggression are prone to social desirability biases (Paulhus, 1991), potentially leading to underestimation. To address this issue, and given the potential biases in youth self‐reports, concerns about teacher reports in the case of FSU immigrant youth (Mirsky, 2012), and known discrepancies between parent and youth reports (Lansford, 2018; Yang et al., 2021), we included both self‐ and parent‐reported measures.

Several limitations should also be acknowledged. First, unlike youth reports, which exhibited individual variability, parent‐reported aggression did not evidence either significant change or variance over time. This limited variance may have constrained our ability to detect broader reciprocal associations with values, as parent‐reported youth aggression was linked only to self‐enhancement values. This discrepancy may, in part, be influenced by differences in measurement tools for youth‐reported aggression and parent‐reported youth aggression, which could capture different manifestations of aggression.

Second, aggression was assessed as a broad construct and was not differentiated into physical versus social/relational forms. This distinction is important for interpreting the observed gender pattern, as prior research indicates that boys tend to exhibit higher levels of physical aggression, whereas girls often display comparable or higher levels of social/relational aggression (Card et al., 2008; Slawinski et al., 2019). Accordingly, the finding that girls were perceived by parents as more aggressive should be interpreted cautiously, in light of evidence that gender differences in aggression depend on aggression subtype. Future research would benefit from assessing aggression subtypes separately to clarify distinct value–aggression pathways.

Third, our measure did not distinguish between proactive and reactive aggression, which may reflect distinct motivational processes. This distinction is relevant among immigrant adolescents, for whom bullying behavior may be primarily driven by affiliation‐related proactive aggression to achieve control in uncertain situations, rather than by reactive, emotionally driven aggression (Fandrem et al., 2009). Future research should employ consistent measurement tools across informants and incorporate additional perspectives from informants who regularly observe youth in social settings, such as teachers (Clemans et al., 2014), as well as include instruments that differentiate between proactive and reactive forms of aggression to better capture their distinct motivational underpinnings.

Fourth, we relied on self‐reported measures of values, the most common method for capturing individuals' own priorities and beliefs. However, recent research (e.g., Ginosar Yaari et al., 2024) has examined interpersonal value perception using dyadic designs, in which individuals (e.g., parents, children, romantic partners) report both their own values and their perceptions of a close other's values, while the other does the same. These studies show that individuals tend to perceive others' values through the lens of their own, often leading to biased or incomplete understanding. Future research could address this limitation by employing multi‐informant approaches that capture each party's perspective, alongside qualitative methods such as interviews that explore how adolescents understand and experience values (Lewis‐Smith et al., 2021).

Another limitation concerns the sample of immigrant adolescents and parents from the FSU in Israel, which may reflect unique characteristics shaped by both their homeland and destination country. For instance, their experiences of cultural dissonance, individualistic traditions rooted in Soviet‐era values, and a historical emphasis on academic success may influence their adaptation processes (e.g., Remennick, 2019). At the same time, challenges in the destination environment, such as peer rejection, difficulties adapting to new social norms, and heightened risk of maladjustment, further complicate their integration (Elizarov et al., 2023; Mirsky, 2012; Ohana et al., in press; Tamir & Ein‐Tal, 2025; Yakhnich et al., 2025). These factors may limit the generalizability of the present findings to other immigrant groups and cultural contexts. Nonetheless, recent work has highlighted the importance of cross‐cultural comparative research with this group to advance developmental and migration theories (Alpysbekova et al., 2025).

Although age and sex were not included as covariates in the main models, supplementary analyses controlling for these variables yielded consistent results. Future research should further explore their role, especially in more culturally diverse immigrant youth populations. Finally, uneven attrition across waves may have reduced power to detect some effects (Little, 2024). We recognize that sample size and attrition may have limited our ability to detect subtle longitudinal associations. Future research with larger samples and more balanced retention across waves is needed to clarify these developmental patterns.

## CONCLUSIONS AND IMPLICATIONS

The present findings have important theoretical conclusions and practical implications. Theoretically, this study builds on Schwartz's basic values theory (Schwartz, 1992, 2012) and previous evidence to emphasize the bidirectional relationship between values and behavior. Although prior research suggests that values can influence aggression, our findings indicate that aggressive behaviors also shape values over time. This dynamic is particularly relevant under environmental stress, such as migration during adolescence.

From an intervention perspective, the results emphasize that a decrease in aggressive behaviors may serve as a foundation for fostering nurturing and caring‐oriented value systems among immigrant youth. As suggested by Benish‐Weisman (2019), behavioral changes often precede value transformations, making interventions that target behavior an effective starting point. Programs incorporating strategies like specific reinforcement or influencing attitudes such as moral judgment (e.g., Aquilar et al., 2018), teaching conflict resolution skills (e.g., communication, active listening, problem‐solving; Li, 2022), and promoting prosocial behaviors through structured sports group activities (e.g., Martinek & Hellison, 2016) have proven effective, particularly for at‐risk youth (e.g., Wright et al., 2016). These interventions can reduce aggression while fostering empathy and social responsibility (Manzano‐Sánchez & Valero‐Valenzuela, 2019), goals that are aligned with self‐transcendence values. In addition, research on behavioral therapy and spiritual rituals supports the notion that repeated enactment of specific behaviors and activities can lead to internal value change (e.g., Cuijpers et al., 2007; Fischer, 2017). By targeting aggression first and gradually fostering self‐transcendence values, interventions can create a cascading effect that reduces aggression, promotes adaptive value systems, and strengthens immigrant adolescents' social connections in their new cultural environment. Future research should explore the long‐term effects of such approaches on social adjustment and resilience among immigrant youth.

## AUTHOR CONTRIBUTIONS

**Mark Van Ryzin:** Formal analysis. **Einat Elizarov:** Investigation. **Adi Arden:** Conceptualization. **Maya Benish‐Weisman:** Writing – review and editing; supervision. **Hanit Ohana:** Conceptualization; writing – original draft. **Seth J. Schwartz:** Writing – review and editing.

## FUNDING INFORMATION

This research was funded by the European Union (ERC, BeValue project, Grant No. 101087000; awarded to Maya Benish‐Weisman). Views and opinions expressed are, however, those of the authors only and do not necessarily reflect those of the European Union or the European Research Council (ERC). Neither the European Union nor the ERC can be held responsible for them. This research was supported by the United States–Israel Binational Science Foundation (BSF, Grant No. 2024057; awarded to Maya Benish‐Weisman and Seth J. Schwartz).

## CONFLICT OF INTEREST STATEMENT

The authors have no conflicts of interest to disclose. Materials and analysis codes for this study are available by emailing the corresponding author.

## ETHICS STATEMENT

This study was approved by the Institutional Review Board (IRB) at the Hebrew University of Jerusalem (Approval Number: 133/20).

## CONSENT

Informed consent was obtained from all participants, including parents and their adolescent children, in accordance with ethical guidelines.

## POSITIONALITY STATEMENT

Although the authors do not belong to the immigrant group under study, they have conducted this research with a commitment to ethical integrity, cultural sensitivity, and methodological rigor. Efforts were made to minimize bias through peer debriefing, the inclusion of both parent and youth reports to ensure a comprehensive analysis, and engagement with literature on immigrant experiences.

## Supporting information


Table S1.

## Data Availability

The data that support the findings of this study are available from the corresponding author upon request.
