# Supplementary material for: Aggression and personal values in immigrant adolescents: A longitudinal examination of reciprocal associations
Source: J Res Adolesc. 2026 Apr 23;36:e70188. doi: 10.1111/jora.70188 (PMC13106107; doi:10.1111/jora.70188)
Supplement: Supplementary file 1 — Table S1. [file JORA-36-0-s001.docx]

**Supporting Information**

| **Table S1**  *Items and CFA Outcomes for Parent-Reported Youth Aggression at T1* | | | | | |
| --- | --- | --- | --- | --- | --- |
| Item | Loading | SE | R^2^ | Error  variance | p-value |
| 1. Disruptive; annoys and bothers others. | .59 | .09 | .35 | .10 | .000 |
| 2. Fights. | .65 | .13 | .42 | .15 | .000 |
| 3. Tries to dominate others; bullies; threatens. | .53 | .16 | .28 | .16 | .001 |
| 4. Teases others. | .63 | .13 | .39 | .16 | .000 |
| 5. Blames others; denies own mistakes. | .53 | .07 | .28 | .07 | .000 |
| 6. Deliberately cruel to others. | .67 | .14 | .45 | .19 | .000 |

*Note.* Standardized results are presented.

**Table S2**

*Items for Youth-Reported Aggression at T1*

| Item |
| --- |
| \| 1. Blamed Others \| \| --- \| \| 2. Tried to hurt someone’s feelings \| \| 3. Made fun of someone behind his/her back \| \| 4. Excluded someone from group activities when angry with him/her \| \| 5. Intentionally damaged someone’s reputation \| \| 6. Tried to turn others against someone when angry with him/her \| \| 7. Gave someone the silent treatment when angry with him/her \| \| 8. Called someone names behind his/her back \| \| 9. Revealed someone’s secrets when angry with him/her \| \| 10. Was rude towards others \| \| 11. Made negative comments about other’s appearance \| |

*Note. The STAB (Burt & Donnellan, 2009) is a validated and widely used measure of youth-reported aggression.*

# **Table S3** *Independent Samples t-tests Comparing Participants Who Remained vs. Dropped Out after T1*

|  | Remained | Dropped |  |  |  |
| --- | --- | --- | --- | --- | --- |
| Variables | M (SD) | M (SD) | t(df) | *p* | *d* |
| Self-Enhancement | 3.80 (0.72) | 3.81 (0.81) | -0.12 (194) | .902 | 0.01 |
| Self-Transcendence | 4.30 (0.51) | 4.30 (0.49) | -0.07 (194) | .948 | 0.0 |
| Openness to Change | 4.46 (0.55) | 4.47 (0.57) | -0.08 (194) | .940 | 0.02 |
| Conservation | 3.46 (0.55) | 3.44 (0.58) | 0.18 (194) | .858 | 0.03 |
| Aggression YR | 1.31 (0.29) | 1.25 (0.22) | 1.48 (178) | .140 | 0.23 |
| Aggression PR | 8.09 (1.68) | 8.39 (2.27) | -1.01 (178) | .316 | 0.16 |
| Youth Gender | 1.41 (0.49) | 1.48 (0.50) | -1.07 (207) | .286 | 0.14 |
| Youth Age | 13.7 (1.30) | 13.6 (1.41) | 0.21 (195) | .837 | 0.08 |
| Youth Grage | 3.32 (1.26) | 3.07 (1.33) | 1.36 (195) | .171 | 0.2 |
| Time in Israel | 2.50 (2.44) | 2.49 (2.48) | 0.25 (68) | .802 | 0.01 |

*Note*: YR = Youth report; PR = parent report on youth aggression; Remained = participants who provided data at T4.

| **Table S4**  *Growth Terms for Youth-Reported Aggressive Behavior* | | | | | | | | |
| --- | --- | --- | --- | --- | --- | --- | --- | --- |
| Model | Values | | | | Aggressive Behavior | | | |
|  | Means | | Variances | | Means | | Variances | |
|  | Intercept | Slope | Intercept | Slope | Intercept | Slope | Intercept | Slope |
| Self-Enhancement | 3.79^***^ (.06) | .32^*^ (.16) | .47^***^ (.06) | .03^**^ (.01) | 1.29^***^ (.02) | .12 (.07) | .05^***^ (.01) | .003^†^ (.002) |
| Self-Transcendence | 4.30^***^ (.04) | .64^**^ (.23) | .17^***^ (.03) | .01 (.01) | 1.29^***^ (.02) | .01 (.14) | .04^***^ (.01) | .002 (.001) |
| Openness to Change | 4.47^***^ (.04) | .52^*^ (.23) | .21^***^ (.03) | .02^*^ (.01) | 1.29^***^ (.02) | .17 (.11) | .04^***^ (.01) | .003^†^ (.002) |
| Conservation Values | 3.45^***^ (.04) | .49^*^ (.20) | .23^***^ (.03) | .01^*^ (.004) | 1.29^***^ (.02) | .07 (.13) | .05^***^ (.01) | .003^*^ (.002) |
| ^†^*p* = .05. ^*^*p* < .05. ^**^*p* < .01. ^***^*p* < .001. | | | | | | | | |

| **Table S5**  *Growth Terms for Parent-Report of Youth Aggressive Behavior* | | | | | | | | |
| --- | --- | --- | --- | --- | --- | --- | --- | --- |
| Model | Values | | | | Aggressive Behavior | | | |
|  | Means | | Variances | | Means | | Variances | |
|  | Intercept | Slope | Intercept | Slope | Intercept | Slope | Intercept | Slope |
| Self-Enhancement | 3.79^***^ (.06) | .34^†^ (.18) | .47^***^ (.06) | .03^**^ (.01) | 8.20^***^ (.15) | .27 (.40) | 2.60^***^ (.38) | .01 (.04) |
| Self-Transcendence | 4.30^***^ (.04) | .64^**^ (.23) | .18^***^ (.03) | .01^*^ (.004) | 8.20^***^ (.15) | .14 (.75) | 2.59^***^ (.38) | .01 (.04) |
| Openness to Change | 4.47^***^ (.04) | .78^***^ (.22) | .22^***^ (.03) | .02^*^ (.01) | 8.21^***^ (.15) | 1.05 (.58) | 2.60^***^ (.38) | .01 (.04) |
| Conservation Values | 3.45^***^ (.04) | .23 (.17) | .23^***^ (.03) | .01^*^ (.004) | 8.21^***^ (.15) | .72 (.53) | 2.58^***^ (.38) | .01 (.04) |
| ^†^*p* = .05. ^*^*p* < .05. ^**^*p* < .01. ^***^*p* < .001. | | | | | | | | |

# **Table S6**

*Correlations Coefficients Between Youth- and Parent-Reported Aggression Across Waves*

|  | Aggression YR (W1) | Aggression YR (W2) | Aggression YR (W3) | Aggression YR (W4) |
| --- | --- | --- | --- | --- |
| 1. Aggression PR (W1) | .01 | .02 | .07 | .10 |
| 6. Aggression PR (W2) | .07 | .09 | .11 | .02 |
| 7. Aggression PR (W3) | −.06 | .11 | .27** | .19 |
| 8. Aggression PR (W4) | .18 | .35*** | .24* | .13 |

*Note.* YR = Youth report; PR = parent report on youth aggression. * p < .05. ** p < .01.

| **Table S7a**  *Correlations, Means and Standard Deviations for Boys* | | | | | | | | | | | | | | | | | | | | | | | | |
| --- | --- | --- | --- | --- | --- | --- | --- | --- | --- | --- | --- | --- | --- | --- | --- | --- | --- | --- | --- | --- | --- | --- | --- | --- |
|  | Self-E | | | | Self-T | | | | Open | | | | Con | | | | Stab | | | | Rbpc | | | |
|  | 1 | 2 | 3 | 4 | 5 | 6 | 7 | 8 | 9 | 10 | 11 | 12 | 13 | 14 | 15 | 16 | 17 | 18 | 19 | 20 | 21 | 22 | 23 | 24 |
| 1. Self-E (W1) | — |  |  |  |  |  |  |  |  |  |  |  |  |  |  |  |  |  |  |  |  |  |  |  |
| 2. Self-E (W2) | .64^***^ | — |  |  |  |  |  |  |  |  |  |  |  |  |  |  |  |  |  |  |  |  |  |  |
| 3. Self-E (W3) | .61^***^ | .73^***^ | — |  |  |  |  |  |  |  |  |  |  |  |  |  |  |  |  |  |  |  |  |  |
| 4. Self-E (W4) | .32^**^ | .65^***^ | .73^***^ | — |  |  |  |  |  |  |  |  |  |  |  |  |  |  |  |  |  |  |  |  |
| 5. Self-T (W1) | -.54^***^ | -.34^***^ | -.33^**^ | -.05 | — |  |  |  |  |  |  |  |  |  |  |  |  |  |  |  |  |  |  |  |
| 6. Self-T (W2) | -.43^***^ | -.58^***^ | -.50^***^ | -.34^**^ | .61^***^ | — |  |  |  |  |  |  |  |  |  |  |  |  |  |  |  |  |  |  |
| 7. Self-T (W3) | -.23^*^ | -.31^*^ | -.51^***^ | -.42^***^ | .51^***^ | .73^***^ | — |  |  |  |  |  |  |  |  |  |  |  |  |  |  |  |  |  |
| 8. Self-T (W4) | -.02 | -.06 | -.20 | -.31^*^ | .41^***^ | .53^***^ | .67^***^ | — |  |  |  |  |  |  |  |  |  |  |  |  |  |  |  |  |
| 9. Open (W1) | .14 | .22^*^ | .09 | .09 | -.36^***^ | -.30^**^ | -.25^*^ | -.04 | — |  |  |  |  |  |  |  |  |  |  |  |  |  |  |  |
| 10. Open (W2) | .02 | .12 | -.03 | .08 | -.23^*^ | -.25^*^ | -.24* | -.09 | .64^***^ | — |  |  |  |  |  |  |  |  |  |  |  |  |  |  |
| 11. Open (W3) | .07 | .004 | .03 | -.07 | -.13 | -.14 | -.21 | .001 | .55^***^ | .56^***^ | — |  |  |  |  |  |  |  |  |  |  |  |  |  |
| 12. Open (W4) | .05 | -.004 | .08 | .12 | -.11 | -.11 | -.18 | -.20 | .47^***^ | .47^***^ | .74^***^ | — |  |  |  |  |  |  |  |  |  |  |  |  |
| 13. Con (W1) | -.54^***^ | -.45^***^ | -.34^***^ | -.34^**^ | -.02 | .10 | -.05 | -.30^*^ | -.67^***^ | -.39^***^ | -.42^***^ | -.33^**^ | — |  |  |  |  |  |  |  |  |  |  |  |
| 14. Con (W2) | -.29^**^ | -.53^***^ | -.24^*^ | -.32^*^ | .03 | -.06 | -.18 | -.31^*^ | -.42^***^ | -.61^***^ | -.29^*^ | -.23 | .61^***^ | — |  |  |  |  |  |  |  |  |  |  |
| 15. Con (W3) | -.33^**^ | -.30^*^ | -.40^***^ | -.22 | -.05 | -.10 | -.18 | -.36^**^ | -.33^**^ | -.27^*^ | -.66^***^ | -.53^***^ | .65^***^ | .58^***^ | — |  |  |  |  |  |  |  |  |  |
| 16. Con (W4) | -.27^*^ | -.40^***^ | -.43^***^ | -.57^***^ | -.19 | -.04 | -.06 | -.31^**^ | -.30^**^ | -.27^*^ | -.39^***^ | -.60^***^ | .63^***^ | .55^***^ | .73^***^ | — |  |  |  |  |  |  |  |  |
| 17. Stab (W1) | .41^***^ | .38^***^ | .31^**^ | .16 | -.25^**^ | -.28^**^ | -.12 | -.02 | .15 | .12 | -.01 | -.09 | -.30^*^ | -.19 | -.13 | -.05 | — |  |  |  |  |  |  |  |
| 18. Stab (W2) | .31^***^ | .47^***^ | .44^***^ | .38^**^ | -.01 | -.23^*^ | -.16 | -.03 | .04 | .17 | -.02 | -.006 | -.27^*^ | -.34^**^ | -.22 | -.25^*^ | .47^***^ | — |  |  |  |  |  |  |
| 19. Stab (W3) | .29^*^ | .30^*^ | .31^**^ | .30^*^ | .07 | -.05 | -.02 | -.03 | -.14 | -01 | -.05 | -.002 | -.17 | -.21 | -.16 | -.17 | .50^***^ | .60^***^ | — |  |  |  |  |  |
| 20. Stab (W4) | .20 | .31^*^ | .28^*^ | .47^***^ | .10 | -.17 | -.19 | -.26^*^ | -.24^*^ | -.07 | -.14 | .04 | -.05 | -.04 | -.04 | -.18 | .40^***^ | .49^***^ | .64^***^ | — |  |  |  |  |
| 21. Rbpc (W1) | -.08 | .06 | .12 | .15 | .06 | -.26^*^ | -.30^*^ | -.02 | -.03 | .01 | .26^*^ | -.19 | .07 | .12 | .29^*^ | .04 | -.04 | .16 | .15 | .16 | — |  |  |  |
| 22. Rbpc (W2) | .08 | .17 | .13 | .09 | -.16 | -.27^*^ | -.21 | .02 | -.29^**^ | -.22 | .06 | .003 | .29^*^ | .06 | .04 | .01 | .12 | .12 | .11 | -.02 | .71^***^ | — |  |  |
| 23. Rbpc (W3) | .06 | .14 | .17 | .11 | .05 | -.13 | -.04 | .05 | -.19 | -.23 | -.23 | -.06 | .11 | .09 | .08 | -.06 | -.08 | .06 | .23 | .09 | .71^***^ | .72^***^ | — |  |
| 24. Rbpc (W4) | .17 | .07 | .18 | .08 | .002 | -.11 | -.13 | .07 | -.14 | -.06 | -.22 | -.14 | .02 | .05 | .13 | -.02 | -.15 | .39^**^ | .27^*^ | .14 | .67^***^ | .62^***^ | .62^**^ | — |
| *N* | 104 | 88 | 73 | 69 | 104 | 88 | 73 | 69 | 104 | 88 | 73 | 69 | 104 | 88 | 73 | 69 | 95 | 84 | 70 | 66 | 97 | 81 | 71 | 68 |
| *M* | 3.75 | 3.79 | 4.03 | 4.00 | 4.32 | 4.30 | 4.15 | 4.32 | 3.51 | 4.42 | 4.43 | 4.37 | 3.50 | 3.50 | 3.48 | 3.54 | 1.26 | 1.28 | 1.22 | 1.28 | 7.74 | 7.90 | 7.83 | 7.69 |
| *SD* | .75 | .78 | .65 | .75 | .48 | .47 | .47 | .49 | .48 | .49 | .54 | .50 | .48 | .47 | .47 | .58 | .24 | .33 | .27 | .32 | 1.19 | 1.41 | 1.51 | 1.34 |
| ^*^*p* < .05. ^**^*p* < .01. ^***^*p* < .001. Stab = Youth-reported aggression, Rbpc = Parent-report youth aggression.  **Table S7b**  *Correlations, Means and Standard Deviations for Girls* | | | | | | | | | | | | | | | | | | | | | | | | |
|  | Self-E | | | | Self-T | | | | Open | | | | Con | | | | Stab | | | | Rbpc | | | |
|  | 1 | 2 | 3 | 4 | 5 | 6 | 7 | 8 | 9 | 10 | 11 | 12 | 13 | 14 | 15 | 16 | 17 | 18 | 19 | 20 | 21 | 22 | 23 | 24 |
| 1. Self-E (W1) | — |  |  |  |  |  |  |  |  |  |  |  |  |  |  |  |  |  |  |  |  |  |  |  |
| 2. Self-E (W2) | .74^***^ | — |  |  |  |  |  |  |  |  |  |  |  |  |  |  |  |  |  |  |  |  |  |  |
| 3. Self-E (W3) | .74^***^ | .79^***^ | — |  |  |  |  |  |  |  |  |  |  |  |  |  |  |  |  |  |  |  |  |  |
| 4. Self-E (W4) | .48^**^ | .65^***^ | .71^***^ | — |  |  |  |  |  |  |  |  |  |  |  |  |  |  |  |  |  |  |  |  |
| 5. Self-T (W1) | -.56^***^ | -.44^***^ | -.43^**^ | -.31^*^ | — |  |  |  |  |  |  |  |  |  |  |  |  |  |  |  |  |  |  |  |
| 6. Self-T (W2) | -.51^***^ | -.58^***^ | -.50^***^ | -.48^**^ | .61^***^ | — |  |  |  |  |  |  |  |  |  |  |  |  |  |  |  |  |  |  |
| 7. Self-T (W3) | -.36^*^ | -.34^**^ | -.59^***^ | -.38^*^ | .68^***^ | .51^***^ | — |  |  |  |  |  |  |  |  |  |  |  |  |  |  |  |  |  |
| 8. Self-T (W4) | -.35^*^ | -.41^**^ | -.55 | -.61^*^ | .55^***^ | .49^***^ | .71^***^ | — |  |  |  |  |  |  |  |  |  |  |  |  |  |  |  |  |
| 9. Open (W1) | .29^**^ | .35^**^ | .49^***^ | .15 | -.29^**^ | -.18 | -.25 | -.17 | — |  |  |  |  |  |  |  |  |  |  |  |  |  |  |  |
| 10. Open (W2) | .32^**^ | .32^*^ | .35^*^ | .11 | -.18 | -.35^**^ | -.05 | .16 | .61^***^ | — |  |  |  |  |  |  |  |  |  |  |  |  |  |  |
| 11. Open (W3) | .19 | .37^*^ | .35^*^ | .21 | -.12 | -.18 | -.29^*^ | -.12 | .56^***^ | .69^***^ | — |  |  |  |  |  |  |  |  |  |  |  |  |  |
| 12. Open (W4) | .18 | .38^*^ | .29 | .42^**^ | .07 | -.24 | -.02 | -.21 | .37^*^ | .54^***^ | .66^***^ | — |  |  |  |  |  |  |  |  |  |  |  |  |
| 13. Con (W1) | -.52^***^ | -.53^***^ | -.61^***^ | -.27^*^ | -.06 | .12 | -.02 | -.04 | -.75^***^ | -.54^***^ | -.45^***^ | -.45^**^ | — |  |  |  |  |  |  |  |  |  |  |  |
| 14. Con (W2) | -.52^**^ | -.63^***^ | -.61^***^ | -.29^*^ | .06 | .04 | -.01 | -.13 | -.59^***^ | -.70^***^ | -.65^***^ | -.50^***^ | .79^***^ | — |  |  |  |  |  |  |  |  |  |  |
| 15. Con (W3) | -.46^***^ | -.60^***^ | -.59^***^ | -.41^**^ | -.05 | .21 | -.05 | -.03 | -.61^***^ | -.67^***^ | -.73^***^ | -.67^***^ | .81^***^ | .86^***^ | — |  |  |  |  |  |  |  |  |  |
| 16. Con (W4) | -.27^*^ | -.49^***^ | -.36^*^ | -.58^***^ | -.16 | .23 | -.16 | -.04 | -.26 | -.53^***^ | -.51^***^ | -.84^***^ | .50^***^ | .62^***^ | .76^***^ | — |  |  |  |  |  |  |  |  |
| 17. Stab (W1) | .33^**^ | .35^**^ | .47^***^ | .18 | -.14 | -.11 | -.24 | -.07 | .04 | .13 | .33^*^ | .25 | -.14 | -.27^*^ | -.40^**^ | -.23 | — |  |  |  |  |  |  |  |
| 18. Stab (W2) | .18 | .24 | .44^**^ | .17 | -.01 | -.09 | -.22 | -.08 | .16 | .20 | .38^*^ | .31^*^ | -.19 | -.27^*^ | -.41^**^ | -.27 | .77^***^ | — |  |  |  |  |  |  |
| 19. Stab (W3) | .09 | .22 | .39^**^ | .34^*^ | .14 | -.002 | -.16 | -.38^*^ | .06 | -.009 | .26^*^ | .43^**^ | -.18 | -.15 | -.33^*^ | -.27 | .52^***^ | .65^***^ | — |  |  |  |  |  |
| 20. Stab (W4) | .25 | .25 | .50^***^ | .28^*^ | -.04 | -.21 | -.35^*^ | -.35^*^ | .19 | .23 | .51^***^ | .45^**^ | -.25 | -.22 | -.46^**^ | -.28^*^ | .50^***^ | .63^***^ | .70^***^ | — |  |  |  |  |
| 21. Rbpc (W1) | .05 | .19 | .19 | .20 | -.16 | -.27^*^ | -.05 | -.36^*^ | .28^*^ | .22 | .17 | .08 | -.11 | -.09 | .21 | .06 | .004 | -.08 | .01 | .10 | — |  |  |  |
| 22. Rbpc (W2) | .08 | .20 | .31^*^ | .24 | -.02 | -.15 | -.07 | -.39^*^ | -.35^**^ | .19 | .22 | -.01 | -.31^*^ | -.20 | -.31^*^ | -.04 | .01 | .05 | .09 | .13 | .64^***^ | — |  |  |
| 23. Rbpc (W3) | .21 | .19 | .35^*^ | .26 | -.05 | -.46^***^ | -.28^*^ | .42^**^ | .09 | .24 | .17 | .17 | -.19 | -.03 | -.22 | -.04 | -.09 | .19 | .34^*^ | .40^**^ | .51^***^ | .70^***^ | — |  |
| 24. Rbpc (W4) | .16 | .26 | .37^*^ | .35^*^ | -.12 | -.24 | -.23 | -.18 | .21 | .04 | .09 | .06 | -.16 | -.10 | -.19 | -.17 | .17 | .32^*^ | .21 | .14 | .22 | .49^***^ | .21^**^ | — |
| *N* | 91 | 64 | 54 | 47 | 91 | 64 | 54 | 47 | 91 | 64 | 54 | 47 | 91 | 64 | 54 | 47 | 84 | 57 | 50 | 46 | 82 | 69 | 58 | 44 |
| *M* | 3.86 | 3.82 | 3.93 | 4.09 | 4.30 | 4.45 | 4.26 | 4.26 | 4.50 | 4.46 | 4.42 | 3.41 | 3.33 | 3.40 | 3.35 | 3.54 | 1.32 | 1.29 | 1.26 | 1.24 | 8.76 | 8.77 | 8.40 | 8.11 |
| *SD* | .76 | .87 | .85 | .70 | .52 | .53 | .57 | 41 | .57 | .53 | .59 | .54 | .61 | .56 | .64 | .58 | .28 | .27 | .29 | .22 | 2.46 | 2.41 | 2.11 | 1.79 |

^*^*p* < .05. ^**^*p* < .01. ^***^*p* < .001. Stab = Youth-reported aggression, Rbpc = Parent-report youth aggression.
